# Elimination of Ethanol for the Production of Fucoidans from Brown Seaweeds: Characterization and Bioactivities

**DOI:** 10.3390/md22110493

**Published:** 2024-10-31

**Authors:** Periaswamy Sivagnanam Saravana, Shanmugapriya Karuppusamy, Dilip K. Rai, Janith Wanigasekara, James Curtin, Brijesh K. Tiwari

**Affiliations:** 1 Department of Food Chemistry & Technology, Teagasc Food Research Centre, D15 DY05 Dublin, Ireland; dilip.rai@teagasc.ie; 2 Department of Biological Sciences, Munster Technological University, Bishopstown, T12 P928 Cork, Ireland; 3 School of Biosystems and Food Engineering, University College Dublin, Belfield, D04 V1W8 Dublin, Ireland; shanmugapriya.karuppusamy@ucd.ie; 4 School of Food Science and Environmental Health, Technological University Dublin, D07 ADY7 Dublin, Ireland; janith.manoharawanigasekara@tudublin.ie (J.W.); james.curtin@tudublin.ie (J.C.)

**Keywords:** seaweeds, fucoidan, molecular weight cut off, FTIR, antioxidant, cytotoxicity

## Abstract

Fucoidan, a sulphated polysaccharide from brown seaweed composed of several monosaccharides, has been stated to have several bioactive properties such as antioxidant, antiviral, anticancer, antithrombic, anti-inflammatory, and immunomodulatory effects. This paper provides research findings on green extraction methods, structural analysis of fucoidan, and its associated bioactivities. Fucoidans from brown seaweeds, *Fucus vesiculosus* and *Ascophyllum nodosum,* were extracted using green solvents such as citric acid (CA) followed by MWCO (molecular weight cut-off) filtration to obtain high-purity polysaccharides. The presence of functional groups typical to fucoidans, namely, fucose, sulfate, and glycosidic bonds, in the extracts were confirmed through the data obtained from FTIR (Fourier-transform infrared spectroscopy), TGA (thermogravimetric analysis), DSC (differential scanning calorimetry), and solid-state CP–MAS (cross-polarization magic angle spinning) analysis. The MWCO analysis identified that the >300 kDa fraction can have better content of fucoidan (FV-CA 79.16%, FV-HCl 63.59%, AN-CA 79.21%, AN-HCl 80.70%) than the conventional extraction process. Furthermore, the >300 kDa fraction showed significantly higher antioxidant activities compared to crude fucoidan extracts. Crude fucoidan extracts showed significant inhibition of cell viability in human lung (A459 lung carcinoma cells) and colorectal adenocarcinoma (Caco-2) cells at higher concentrations. The fucoidan extracted with green solvents and avoiding alcohol-based precipitation has substantial antioxidant/antitumor action, so, due to this activity, it can be employed as functional foods in food applications.

## 1. Introduction

Brown seaweeds widely found in the Atlantic Ocean possess various type of bioactive compounds, including fucoxanthin, fucoidan, laminarin, peptides, mannitol, and polyphenolic compounds [1,2,3]. These bioactive compounds demonstrate a various type of bioactivities such as antimicrobial, antioxidant, antifungal, antiviral, anticancer, anticoagulant, antidiabetic, anti-HIV, anti-inflammatory, etc. [1]. The key element of the brown seaweed is polysaccharides, which are mainly three types of biopolymers, i.e., alginate, fucoidan, and laminarin [2,3]. Among these, fucoidan is the major biopolymer, which is widely used as a bioactive compound for various applications. Fucoidan is largely composed of fucose and its sulfated forms, and the later form is associated with biological properties of fucoidans [4,5]. The chemical content, including fucoidans, in brown seaweeds can be influenced by seasonal variations, geographic origination, biotic or abiotic factors, etc. [6]. 

The classical extraction method of fucoidan is generally processed using organic solvents containing hydrochloric acid (HCl) or other organic acids with temperature ranging from 70 to 80 °C, followed by calcium chloride (CaCl_2_) treatments and precipitation through alcohol [7]. Fucoidans can be extracted using various eco-friendly technologies such as enzymes, ultrasound, microwave, and subcritical water-based extraction routes [8,9]. However, the operating parameters play a key role in the extraction, structure, and bioactivity of the obtained fucoidan [8,10].

The molecular weight of fucoidan determines it achieving specific bioactivity; however, the molecular weight of this polysaccharide property cannot determine the bioactivity, since it has been involved with many factors of its chemical composition. For instance, a fucoidan with a 5 and 37 kDa shows high antioxidant and cytotoxicity against tumour cell lines [11,12]. Wide-ranging molecular weight (0.8–270 kDa) of fucoidan suggests it to have a reactive oxygen species (ROS) and inhibit the UVB-induced cell death [13], while low-molecular-weight fucoidan (12–760 Da) exhibits in vitro and in vivo effects on osteoporosis [14]. High molecular weights ranging from 490 to >1000 kDa were exhibited for nitric oxide radical scavenging [15], and for antitumoral [16] or anti-inflammatory activities [17]. Anticoagulant properties can be achieved with low-molecular-weight fucoidans (1.4–3.7 kDa) from *Undaria pinnatifida* [18]. Similarly, low-molecular-weight fucoidan (0.817 kDa) from *Saccharina japonica* enhanced and decelerated the growth and development of diabetic nephropathy in rats [19] and *Sargassum hemiphyllum*-based low-molecular-weight fucoidan (0.8 kDa) was proposed as a supplementary treatment to chemotherapy agents with colorectal cancer patients [20]. Fucoidan with 15–20 kDa and 30 kDa can exhibit a proangiogenic effect on human endothelial cells, and from a study it was suggested that fucoidans ranging from 20–30 kDa would be the ideal choice for a proangiogenic effect [21]. At the same time, low-molecular-weight fucoidans have several advantages such as improving bioavailability, higher anticoagulant activity, higher degree of bioactivity, etc. [22]. The low-molecular-weight fucoidan can be produced through hydrolysis without changing its structure with the help of enzymes, acids, ultrasound, radiations, subcritical water, etc. [23,24,25], though it can be achieved concurrently during extraction [26]. Fucoidans obtained from *Sargassum muticum* have yielded widely distributed molecular weights (1–80 kDa) with the optimized extraction conditions from hydrothermal processing [26]. Laminarin is a type of polysaccharide in the brown seaweed; it is a type of storage glucan and it is made of D-glucose with β-(1,3) linkages and with β-(1,6) intrachain branching with a molecular weight range of 5 kDa or less [27].

Fucoidan has been used as functional food in Asia, where its consumption for a long period reduced the risk of cancers [28]. Since the 1980s, fucoidan has been explored for anticancer activities. Numerous scientific reports suggest that fucoidan may prevent metastasis or tumour cell proliferation by inducing cell inhibition angiogenesis and apoptosis [29]. Fucoidan extracts obtained from *Sargassum horneri*, *Eclonia cava*, and *Costaria costata* have been shown to inhibit colon cancer and human melanoma cells [30]. Likewise, fucoidan has exhibited anticancer activity against the lung cancer cell line (A459), where the researchers found that the anticancer activity of fucoidan occurred through signalling pathways such as ERK1/2, Akt-mTOR, and NF-κB [31]. Prior to this experiment, from our lab, we used a fucoidan extract from Irish *Fucus vesiculosus* with MWCO and its fractions for antimicrobial (*B. subtilis*, *E. coli*, *L. innocua,* and *P. fluorescens*) and anticancer (human glioblastoma cell line, U-251 MG) properties [32]. In a similar process related to this work, fucoidans extracted from *F. vesiculosus* were fractionated through MWCO and its effects were studied against rotavirus and food borne pathogens [33].

The objective of the current research was to obtain fucoidan from two different brown seaweeds (*A. nodosum* and *F. vesiculosus*) through HCl and green solvents such as citric acid (CA), and without the alcohol (ethanol)-based precipitation. Instead, membrane-based separation is deployed to obtain fractions of different molecular weights and assess their anticancer activities against human lung carcinoma (A549) and human colorectal adenocarcinoma cell lines (Caco-2) as well as antioxidant potentials. The chemical characterization of fucoidan extracts was achieved using HPLC-RI, FTIR spectroscopy, TGA, DSC, and solid-state NMR spectroscopy. 

## 2. Results and Discussion

**Figure 1:**
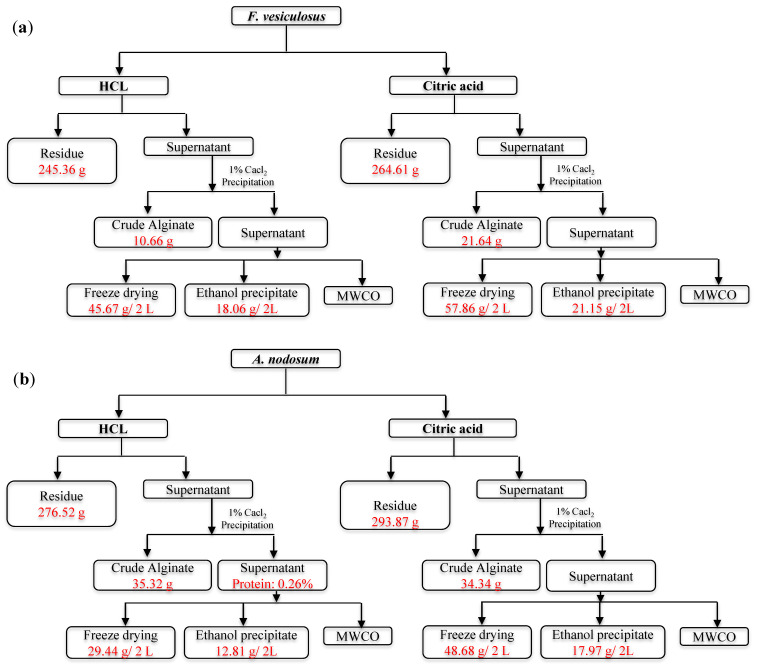
Workflow for the fucoidan production from (**a**) *F. vesiculosus* and (**b**) *A. nodosum.*

The production of fucoidan, a sulfated polysaccharide from selected brown algae including *A. nodosum* and *F. vesiculosus,* was performed as a step-by-step process through the biorefinery approach, as outlined in Figure 1. 

### 2.1. Fucoidan Extraction and Its Chemical Composition

The selected two brown seaweeds were extracted with food-grade acids in a large-scale process to obtain fucoidan. A further separation process was carried out for the fucoidan (Figure 1), including freeze-drying, ethanol precipitation, and MWCO. The lyophilized forms of all the fractions obtained were stored in room temperature in tight container for further analysis studies. 

The chemical compositions of fucoidan extracted from selected two seaweeds, *F. vesiculosus* (Table 1) and *A. nodosum* (Table 2), were determined. The chemical compositions analysed included fucoidan content, total glucan content, total protein content, and molecular weight of the fucoidans. 

Firstly, the results showed that the crude fucoidan (CF) from *F. vesiculosus* (FV) with ethanol precipitation process with hydrochloric acid (HCl) as solvent (FV-HCL-CF) yields a fucoidan content greater than citric acid (CA) as solvent used (FV-CA-CF), which was also a trend in *A. nodosum* (AN) samples (i.e., AN-HCL-CF>AN-CA-CF). The fucoidan content was 85.71% in FV-HCL-CF (Table 1) and 81.20% in AN-HCL-CF (Table 2). Secondly, the freeze-dried fucoidan without ethanol precipitation process showed greater fucoidan content in FV-HCL-FD sample than in the FV-CA-FD sample in FV, whereas AN showed greater fucoidan content in AN-CA-FD than AN-CA-HCL. The fucoidan content was found to be 40% in FV-HCL-FD (Table 1) and 14.59% in AN-CA-FD (Table 2). Finally, the MWCO samples showed higher fucoidan content of 79.16% in FV-CA > 300 kDa and 63.59% in FV-HCL > 300 kDa than the 100, 50, 10, and 5 kDa FV fractions (Table 1). In the AN, a higher fucoidan content was present in the >300 kDa fractions, where AN-HCL > 300 kDa showed 80.70% and the AN-CA > 300 kDa showed 79.21% (Table 1 and Table 2). The chemical compositions of sulphated polysaccharide fucoidan extracted from selected seaweeds were changed by various aspects such as seasonal harvesting of these seaweeds and isolation of fucoidan using various methods [34,35] and affecting their properties [3,32,36]. The fucoidan contents based on the fucose content in the samples were measured and compared. The results showed that crude fucoidan has higher content than FD and MWCO samples due to the solubility nature of fucose units [37], the solvent extraction used [3], and the molecular weight [32]. 

The total glucan content in the various extracts is expressed in mg total glucans/100 mg dried extract (Table 1 and Table 2). In the *F. vesiculosus*, the FV-CA > 300 kDa fraction (4.23 ± 0.19 mg) contained a higher total glucan than the FV-HCL-CF fraction (3.61 ± 0.30 mg). A comparable tendency was detected in the *A. nodusum*, where the AN-CA > 300 kDa contained 3.37 ± 0.07 mg with respect to AN-CA-CF (0.82 ± 0.08 mg). However, different MWCO fractions and the solvent treatment gave different total glucan contents. This may be because the total glucan accounts for laminarin and β-glucans in brown seaweeds [37,38,39]. Thus, in additional analysis of the β-glucan content, the crude extracts showed higher β-glucan content than the MWCO fractionated extracts [34].

An LECO FP628 analyser was used to quantify the protein content in the obtained samples. The results showed that the crude fucoidans in both seaweeds with different treatments showed a lower amount of protein content. However, the MWCO samples showed an altered result compared to the glucan content. The protein contents were found to be higher in the <300 kDa samples for the both the seaweeds (*F. vesiculosus* and *A. nodosum*). However, the lower the kDa cut-off was, the more the protein content reduced. This means the proteins are precipitated in the <300 kDa samples. Among the solvents, CA gave a better yield of protein than the HCL. The contents of protein were higher in the FV-CA < 300 kDa, with 2.96 ± 0.056%, and AN-CA < 300 kDa, with 4.70 ± 0.04%, as presented in Table 1 and Table 2. Results of recent studies predicted that high temperature combined with acids might improve the protein yields in brown seaweeds [34,36,40] due to the ionic interactions between polysaccharide and protein in the cell matrix and the cell wall [1,24,41]. Excessive exposure to sunlight resulted in high nitrogen metabolism in brown seaweeds, leading to increased consumption. However, less nitrogen for protein synthesis resulted in decreased protein content in selected seaweed species. The variation in protein content is due to the extraction method used in the study [2,34].

HPLC-RI was utilized to reveal the distribution of molecular weights of fucoidans based on the retention time. The molecular weights of different extracts in *F. vesiculosus* were as follows: FV-CA-CF with 77.30 kDa, FV-CA-FD with 85.09 kDa, and FV-CA > 300 kDa with 68.88 kDa. Similar molecular weights were observed in *A. nodosum* samples: AN-CA-CF with 82.14 kDa, AN-CA-FD with 102.13 kDa, and AN-CA > 300 kDa with 73.49 kDa. The variation between solvents seems to have a major impact on the molecular weight. For instance, HCl as an extractant solvent gave a slightly low molecular weight fucoidans in crude and >300 kDa fractions (Table 1 and Table 2). However, for the <300 kDa fractionated extracts, the MW was not detectable, indicating much lower molecular weight of fucoidans and a hygroscopic nature in these samples. Between the two seaweeds, *A. nodosum* had a higher MW than the *F. vesiculosus*. The contents of fucoidan, total glucan, protein, and molecular weight determination were significantly different between each sample and molecular weight fraction [21,33,42,43]. This may be due to the different structures of selected seaweed species [34,36]. Based on the results obtained in a chemical composition study, the collection and selection of seaweed based on environmental factors and extraction method were analysed and identified for extracting/processing and targeted synthesis to obtain certain groups of components for further studies [24,44].

### 2.2. Characterization of Fucoidans

#### 2.2.1. FTIR

**Figure 2:**
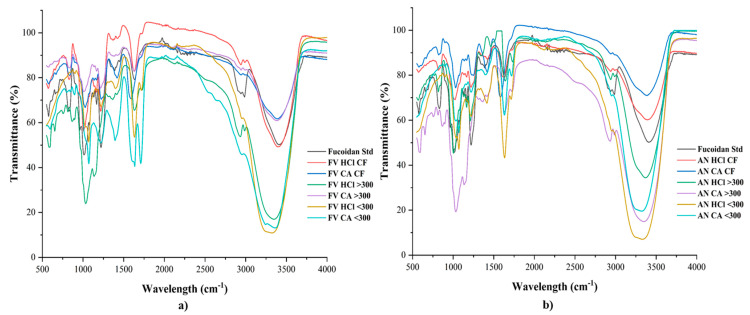
FTIR spectra of fucoidan samples obtained by various extraction and fractionation methods of (**a**) *F. vesiculosus* and (**b**) *A. nodosum*.

The FTIR spectra of various samples obtained from *F. vesiculosus* and *A. nodosum* by the extraction and separation processes are shown in Figure 2a,b. The absorption spectra bands of all samples are presented in the FTIR results. Specifically, seaweed polysaccharides showed the two unique bands in the range of 2000–3600 cm^−1^ region and proved to be evident for the presence of seaweed bioactives in the sample. This band indicates a broad band at 3356 cm^−1^ for the OH and H_2_O stretching vibrations and small bands at 2939, 2941, and 2991 cm^−1^ for the CH stretching with pyranoid ring, and C-6 groups of fucose units. A similar band of 2164 cm^−1^ was observed for fucose units [45]. An absorption peak at around 1730 cm^−1^ was present and related to CO stretching vibration, implying the presence of fatty acids in the *F. vesiculosus*. It indicates the presence of some O-acetyl groups for fucoidans from seaweed [37,46]. A peak in the region of 1628 cm^−1^ was observed and considered to be specific for amide-II [47]. Additionally, it could be related to uronic acid content. A broad band in the range of 1219–1261 cm^−1^ indicates the presence of sulfate ester groups (S=O). This is considered a characteristic feature of fucoidans [23]. A band at 1032 cm^−1^ was observed; this is due to the guluronic and mannuronic acid present as residual alginate content in the samples. In addition, this peak is assigned to C–O–H vibrations of the sugar residues, leading to the presence of monosaccharides and polysaccharides in the samples [22,45]. A sulfate group (C–O–S) was present at the absorption peak of 845 cm^−1^. This band indicates that the sulfate group is at the C-4 position of the fucopyranosyl residue, whereas 823 cm^−1^ could be located at the C-2 and C-3 positions [23,46]. These bands in the FTIR spectra confirm that most of the sulfate groups in these fucoidans are present on the C-4 of fucose units. The sulfate group in the fucoidan structure was responsible for most of the bioactivity. The C-2 sulfate is considered as a structural feature of the fucoidan from *A. nodosum* [21,42]. Additionally, the C-3 sulfate of fucose units was trustworthy for the biological activities of fucoidans [46,47,48].

#### 2.2.2. TGA/DSC

**Figure 3:**
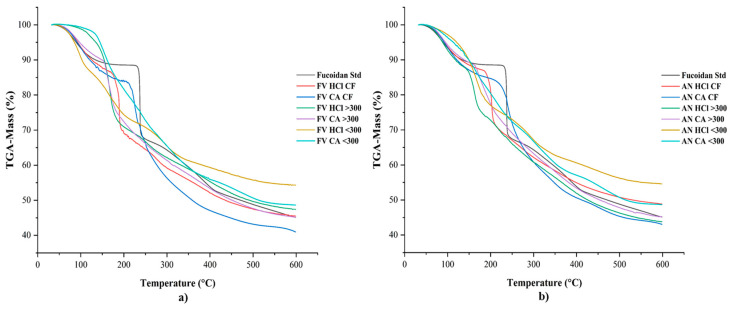
TGA thermograms of fucoidan samples of (**a**) *F. vesiculosus* and (**b**) *A. nodosum* obtained in various conditions.

**Figure 4:**
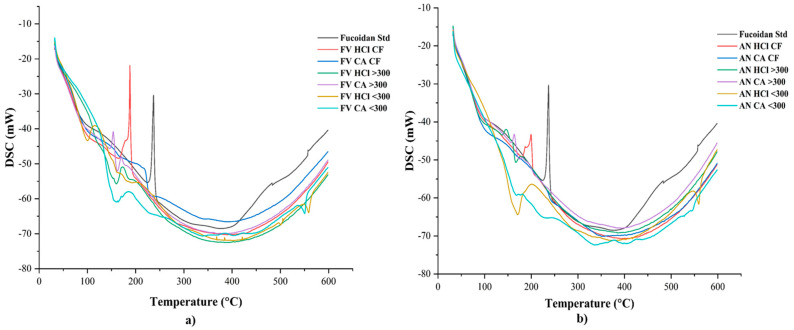
DSC thermograms of fucoidan samples of (**a**) *F. vesiculosus* and (**b**) *A. nodosum* obtained in various conditions.

TGA and DSC thermograms of fucoidan samples obtained from selected seaweeds on various conditions were measured by using TGA analysis. The thermal behaviour of the fucoidan samples over temperatures ranging from 25 to 600 °C were observed and are shown in Figure 3a,b. The TGA graphs of the fucoidan samples obtained from both seaweeds had similar characteristic features in general. The major changes were observed at three stages of degradation at different degradation rates due to the random collapse of glycosidic bonds [25,45,48]. The TGA curve of fucoidan had disappeared due to heating at 100 °C, mainly by the evaporation of water and desorption of water in the samples at 175 °C. The significant weight loss corresponding to degradation rates of the samples was measured based on slow and stable mass loss observed. This stage is considered to be mainly based on the thermal composition of bioactive compounds with strong chemical bonds [3]. The DSC graphs of fucoidan samples are shown in Figure 4a,b. An exothermic transition at 175 °C was measured in the samples and indicates the decomposition or oxidative degradation in the samples [25,45]. The results of the TGA and DSC suggest that the fucoidan extracts have similar degradation nature; however, when compared with the sigma fucoidan, the stability of the standard seems to be higher than the extracted fucoidan samples.

#### 2.2.3. Solid-State ^13^C NMR Spectroscopy

**Figure 5:**
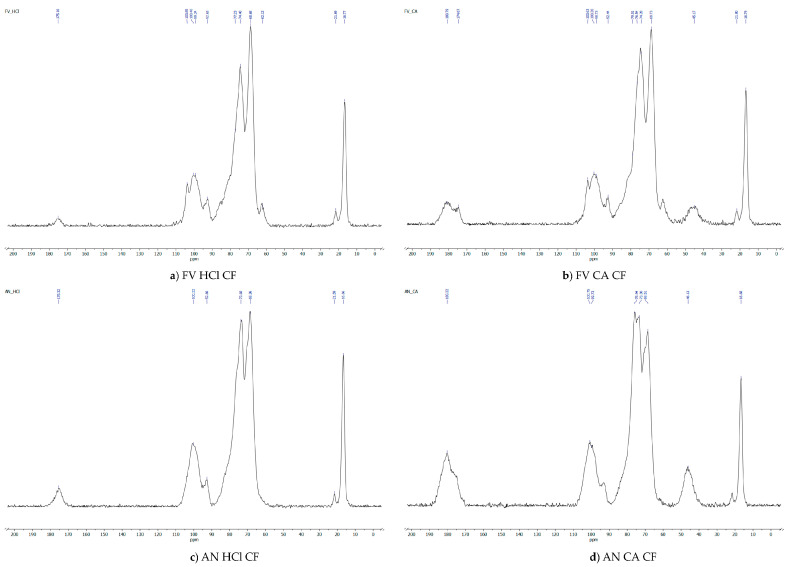
Solid-state CP–MAS spectra of crude fucoidan of (**a**,**b**) *F. vesiculosus* and (**c**,**d**) *A. nodosum*.

The solid-state CP–MAS spectra of crude fucoidan from *F. vesiculosus* and *A. nodosum* were analysed, and the spectra of ^13^C-nuclear magnetic resonance (NMR) are shown in Figure 5. According to NMR spectroscopy data, the characteristics spectra of fucoidan obtained from *F. vesiculosus* and *A. nodosum* were reported. AN-HCl-CF has a linear chain of (1,3)-linked α-L-fucopyranose residues (100.80 and 92.67 ppm) with sulfate groups at positions 2 (73.34 and 63.62 ppm) and position 3 (21.54 and 16.59 ppm), whereas similar spectra were observed in FV-HCl-CF and FV-CA-CF. However, the spectra in AN-CA-CF showed a linear chain of (1,3)-linked α-L-fucopyranose residues (100.62 and 92.92 ppm) with sulfate groups at positions 2 (75.71, 73.45, and 68.55 ppm), position 3 (46.28), and position 4 (21.65 and 16.61 ppm). From the results obtained, the fucose residues were observed to be branched residues and sulfate groups to be substituted at C-2 or C-4 in (1,3)-linked α-L-fucopyranose residues and C-3 in (1,2)- ad (1-4)-linked α-L-fucopyranose residues [4,49]. The anomeric resonances are maintained by intensity of signal from NMR spectra due to the structure of polysaccharides [16,46]. 

### 2.3. Biological Activities of Fucoidan 

#### 2.3.1. Antioxidant Activity

The antioxidant properties of the fucoidan samples obtained from *F. vesiculosus* and *A. nodosum* were evaluated using DPPH (2,2-diphenyl-1-picrylhydrazyl) and ferric reducing antioxidant power (FRAP) assays (Table 3). Fucoidan has been shown to have diverse biological activity. The DPPH and FRAP free radical scavenging activity was determined at 10 mg/mL for all the samples, and we evaluated its antioxidant effects. The FD samples were hygroscopic in nature so the CF and MWCO samples were studied for antioxidant analysis. The DPPH values of the fucoidan ranged from 61.99% to 80.28%. Among the solvent extraction, there is no big difference in the values. However, the contents were slightly altered in the seaweed. *A. nodosum* extracted samples had a higher content of DPPH than the *F. vesiculosus*. However, standard fucoidan (84.91% and vitamin C (77.64%) showed a high percentage inhibition of DPPH antioxidant activity. In the FRAP assay, values of extracts ranged from 2.65 to 9.97 μM Trolox equivalents per mg extract (Table 3), and the standard fucoidan showed a high percentage inhibition of FRAP activity of 11.49 μM Trolox equivalents per mg extract. In the FRAP assay, there was no variation in the solvents-based extraction, and the results were similar to DPPH. However, the major finding was that the MWCO showed a higher content of FRAP in both the seaweeds when compared with the crude fucoidans. FRAP assay influences the antioxidant content during the reducing power assay, evaluating the alteration of Fe^3+^ to Fe^2+^ due to the fucoidan content in the samples. The DPPH antioxidant activity exhibited a stronger effect due to the presence of polyphenols in crude samples [23,43,50] and exhibited a correlation with molecular weight of the fractions [34,37]. The results showed that the activity was mainly owing to the occurrence of sulfate content, type of structural position, functional groups (hydroxyl, sulfate, amine, glycosidic linkages, phosphate, etc.), molecular weight, and other factors, such as impurities of polyphenols, protein, and monosaccharide contents in the samples [25,49]. It may also be possible to recognize the existence of hydrogen atoms from monosaccharide derivatives and their side chain linkages [3,50]. In addition, the scavenging effects of fucoidan samples from *F. vesiculosus* and *A. nodosum* increased with concentration in the present study. 

#### 2.3.2. Cytotoxicity Assay

**Figure 6:**
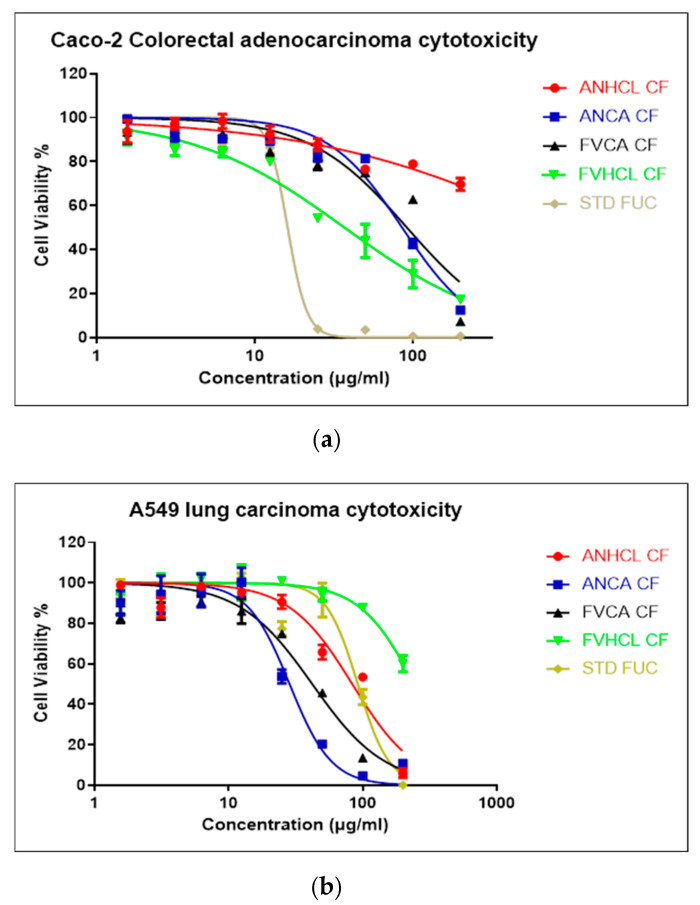
Dose–response curves of cytotoxic activity of crude fucoidan in a concentration range of 1.5625–200 μg/mL against different kinds of cancerous (Caco-2 (**a**) and A549 (**b**)) cell lines.

The cytotoxicity effects of the fucoidan samples obtained from *F. vesiculosus* and *A. nodosum* on cancer cell lines are reported in Figure 6. The dose–response curves of cytotoxic activity of fucoidan samples in a different concentration against different types of cancerous (Caco-2 and A549) cell lines were evaluated in the present study. The effects of fucoidan samples on A549 human lung carcinoma and Caco-2 human colorectal adenocarcinoma cell lines were studied under different concentration gradients (from 200 µg/mL to 1.5625 µg/mL), and cells were post-incubated for 6 days at 37 °C. The different samples in this assay exhibited varying IC_50_ values when tested against A549 human lung carcinoma cells, as follows: AN-HCl-CF (86.57 µg/mL), AN-CA-CF (29.49 µg/mL), FV-CA-CF (43.51 µg/mL), FV-HCl-CF (258.9 µg/mL), and STD FUC (94.48 µg/mL). Meanwhile, the IC_50_ values for the samples when treated with Caco-2 human colorectal adenocarcinoma cells were as follows: AN-HCl-CF (2323 µg/mL), AN-CA-CF (94.11 µg/mL), FV-CA-CF (105.7 µg/mL), FV-HCl-CF (31.23 µg/mL), and STD FUC (15.53 µg/mL). These samples exhibited cytotoxicity effects on both cell lines, with increasing concentrations of fucoidan correlating to greater cytotoxicity. Fucoidans comprise sulfate groups, and their low MW and phenolic content could exhibit strong antiproliferative activity against Caco-2 and A549 cells [23,25,28,51]. The obtained results of fucoidan samples suggest the induction of cell death in specific normal cells or a decline in the rate of cell division [33,51]. Specifically, the viability and proliferation of fucoidan samples depend on the ability of fucoidan to induce apoptosis in cancer cells, leading to a loss of viability [36,43]. 

## 3. Materials and Methods

### 3.1. Materials

*A. nodosum* and *F. vesiculosus* were harvested in March 2019 in Galway Bay Leitir Mór, Connemara, Co., Galway, Ireland. The processing of the seaweed was carried out as described previously [34]. Standards and chemicals, including commercial fucoidan from *F. vesiculosus* (F8190, Sigma, Tokyo, Japan) ≥ 95% purity, ethanol, citric acid, hydrochloric acid, DPPH, 2,4,6-tripyridyl-s-triazine (TPTZ), and 6-hydroxy-2,5,7,8-tetramethylchromane-2-carboxylic acid (Trolox), were purchased from Merck (Wicklow, Ireland). 

### 3.2. Fucoidan Extraction Process

#### 3.2.1. Classical Extraction Process with Acid and Food-Grade Acid

Two brown seaweeds (*F. vesiculosus*, *A. nodosum*) were utilized to obtain fucoidan using a large-scale process. The extraction processes were developed by the Biotechnology company Nutramara, Ireland and are not disclosed here. Following that, extracts were centrifuged for 30 min at 8000 rpm at 4 °C using a Sorvall Lynx 6000 centrifuge (Fisher Scientific Ireland, Dublin, Ireland). Then, the supernatants were further filtered using a muslin cloth to remove any big particles. The filtered supernatants were then mixed with 1% CaCl_2_ (1:1 *v/v*). This solution was kept at 4 °C overnight, and further separation of the extract was carried out by centrifugation with same conditions as mentioned above. The extract was freeze-dried as mentioned in Section 3.3.1 for the mass balance, and the resulting supernatant was divided further to separate fucoidan using different processes. The experimental design is summarized in Figure 1.

### 3.3. Separation Process for Fucoidan

#### 3.3.1. Freeze Drying

From the previous Section 3.2.1, 2 L of the supernatant, which is rich in fucoidan, was freeze-dried in the L-300 Continuous Pro Modular, Buchi, Switzerland (conditions: 30 °C, 0.1 mbar, 48 h). The resulting powder was weighed and stored at −20 °C for further chemical analysis.

#### 3.3.2. Ethanol Precipitation

From the previous Section 3.2.1, 2 L of the supernatant, which is rich in fucoidan, was added to absolute ethanol (3 times the volume of the supernatant) and then it was stored at 4 °C overnight and centrifuged with the same conditions (30 min at 8000 rpm with 4 °C) as mentioned earlier to obtain crude fucoidan. Then, it was freeze-dried as per the abovementioned conditions.

#### 3.3.3. Membrane Based Separation (MWCO)

From the previous Section 3.2.1, 2 L of the supernatant, which is rich in fucoidan, from different types from seaweeds/acids, was subjected to MWCO fractionation using an Amicon^®^ Stirred Cell (Millipore Corporation, Burlington, MA, USA). Sequential MWCO processing was performed using 300 kDa, 100 kDa, 50 kDa, and 10 kDa membranes, similar to the earlier work from our lab [32]. The fractions collected from MWCO were freeze-dried and stored at −20 °C until the next step.

### 3.4. Chemical Composition Analysis of the Fucoidans

#### 3.4.1. Fucoidan Content

The HPLC analysis was carried out to identify the fucoidan content in the obtained samples. The instrument used for this purpose was HPLC-RI, the SUPELCOGEL column, and a flow rate of 0.5 mL/min with 0.1% phosphoric acid as solvent. All the conditions were the same as described in our previous work [32]. A standard curve was developed using the commercial fucoidan. All the analyses were performed in triplicate [34].

#### 3.4.2. Total Glucan Content

The total glucan content in the obtained samples was estimated via the kits protocol K-YBGL (Megazyme, Bray, Ireland). 

#### 3.4.3. Total Protein Content

LECO FP628 (LECO Corp., MI, USA) was used to measure the protein content in the fucoidan samples, and we used a protein conversation factor of 6.25 [45]. 

#### 3.4.4. Molecular Weight

The fucoidan samples obtained in this work were estimated for their molecular weights using an HPLC system using our earlier method [32]. The HPLC carbohydrate column (Shodex, Tokyo, Japan) was used to separate the sample with a mobile phase of NaCl (0.1% *w/v*), and the flow rate was 0.5 mL/min with a temperature of 40 °C. A standard molecular weight (Pullan standard from Sigma) was used to determine the molecular weight of fucoidan samples. All the analyses were carried out a minimum of three times. 

### 3.5. Characterization of the Fucoidan

#### 3.5.1. FTIR Analysis

Each sample extract was grounded into powder, and 0.4 g of each sample extract was compressed into a pellet (with a diameter of 1.4 cm) for FTIR spectra analysis. The Nicolet™ iS5 (FTIR), Thermo Scientific, Madison, WI, USA, was used with diamond crystal attenuated total reflectance (iD7 ATR, Thermo Scientific, Madison, WI, USA). A wavelength of 600–1800 cm^−1^ was used to obtain the signals for all the samples with a resolution of 2 cm^−1^. The tablets prepared for this experiment were scanned at least six times in various surface areas to predict the signals. 

#### 3.5.2. TGA and DSC

TGA and DSC of the samples obtained using optimized extraction conditions were performed using the TGA Q500 instrument (TGA/DSC 3+, Mason Technology, Dublin, Ireland). The nitrogen flow rate was kept at 30 mL/min, with temperature from 25 to 600 °C at a rate of 10 °C/min (constant heating rate).

#### 3.5.3. Solid-State NMR Spectroscopy

The ^13^C (CP-MAS) spectra were recorded using a Bruker 400 WB Plus spectrometer. Chemical shift values are given in ppm.

### 3.6. Biological Activities 

#### 3.6.1. Antioxidant Activities

##### DPPH Assay

The DPPH assay was performed through our earlier work [25]. The sample concentration of this experiment was 1 mg/mL. A 500 µL ascorbic acid or sample was added in an Eppendorf tube and an equal volume of DPPH (100 µM) was added. Then, it was vortexed well and left in the dark for 30 min at room temperature, and the OD value was observed at 515. Then, the results were converted to percentage.

##### FRAP Assay

The FRAP assay was performed according to our earlier publication [48]. The antioxidant activity was expressed in µM Trolox equivalents per mg of extract. For this assay, 20 µL of sample with a similar concentration to DPPH assay was used, and a 280 µL of FRAP working solution was added, then the mixture was vortexed and incubated at 37 °C for 30 min.

#### 3.6.2. Cytotoxicity Assay

##### Cell Culture

The human lung carcinoma (A549) and human colorectal adenocarcinoma cell line (Caco-2) were purchased from ATCC European Distributor (LGC Standards). Dulbecco’s Modified Eagle’s Medium: high glucose (Sigma) was used as a media to cultivate the cells with 10% fetal bovine serum and 1% penicillin–streptomycin (Sigma). The cell was grown at 37 °C in a CO_2_ incubator, and the density of the cell was measured for 2.5 × 10^3^ cells/well. Fucoidan extracts (200 µg/mL) were added to each well in a concentration varying from 1.5625 to 200 µg/mL. A positive control (20% DMSO) and a negative control (media) were maintained throughout the experiment. 

##### Cell Viability Assay

The assay was investigated against Caco-2 and A549 cell lines after being treated with samples using standard cell viability assay using Alamar Blue™ reagent (Thermo Fisher Scientific). The treated cells were washed using PBS, then 10% Alamar Blue™ and 90% DMEM-high glucose solution was added, then they were incubated at 37 °C for 3 h. The cell OD was observed using a Varioskan Lux multiplate reader (Thermo Scientific) with a wavelength of 530 nm (excitation) and 590 nm (emission). The assay was performed with a minimum of three independent tests.

### 3.7. Statistical Analysis

The chemical content and biological assay investigation in this work were conducted thrice, and results are expressed as means ± standard deviation. SPSS (Vision 24, IBM, Armonk, NY, USA) was used to perform statistical analysis, and a one-way ANOVA analysis was performed with Tukey’s HSD post hoc test with a *p*-value of 0.05. The cytotoxicity study of seaweed samples was evaluated using curve fitting and statistical analysis using Prism version 9.1.0, GraphPad Softwares, Inc., La Jolla, CA, USA. The significance level was determined at *p* < 0.001. 

## 4. Conclusions

A cleaner and greener route to extraction and fractionation of fucoidan from two different brown seaweeds (*A. nodosum* and *F. vesiculosus*) was proposed. MWCO fractions using MWCO membranes yield fucoidan content (FV-CA > 300 kDa has 79.16%, FV-CA crude has 71.57%, AN-CA > 300 kDa has 79.21%, and AN-CA crude has 59.82%) significantly higher than or similar to the crude extracts. Furthermore, the chemical characteristics of the obtained fucoidans from MWCO have a similar composition to the commercially available fucoidans. The DPPH activity of the samples ranged from 60.97% to 80.28% and the FRAP ranged from 2.65 to 9.97 μM Trolox equivalents per mg extract. The crude fucoidan exhibited cell viability against Caco-2, with IC_50_ values ranging from 31.23 µg/mL to 2323 µg/mL, and A459 lung carcinoma cells with IC_50_ values ranging from 29.49 µg/mL to 258.9 µg/mL, as equal to the commercial fucoidan (IC_50_ values for Caco-2 were 15.53 µg/mL and A459 was 94.48 µg/mL). Hence, this method reveals a process to produce fucoidan through a clean, alcohol-free separation and an effective extraction process from seaweed materials rich in bioactivity.

## Figures and Tables

**Table 1:** Chemical composition of fucoidan extracted from *F. vesiculosus.*

Fucoidan Obtained from Various Process	Sample	Fucoidan Content (%)	Total Glucan (mg Total Glucans/ 100 mg Dried Extract)	Protein (%)	Molecular Weight (kDa)
Crude fucoidan (with alcohol precipitation)	FV-CA-CF	71.57 ± 2.54 ^b^	2.43 ± 0.23 ^c,d^	0.23 ± 0.01 ^d^	77.30 ± 1.30 ^b^
	FV-HCL-CF	85.71 ± 0.17 ^a^	3.61 ± 0.30 ^a,b^	0.22 ± 0.00 ^d^	33.54 ± 0.36 ^e^
FD_fucoidan (without alcohol precipitation)	FV-CA-FD	16.37 ± 0.25 ^g,h,i^	2.24 ± 0.14 ^c,d^	0.38 ± 0.02 ^d^	85.09 ± 5.92 ^a^
	FV-HCL-FD	40.00 ± 0.63 ^d^	3.29 ± 0.30 ^b,c^	0.45 ± 0.02 ^d^	2.27 ± 0.00 ^f,g^
MWCO	FV-CA > 300 kD	79.16 ± 2.15 ^b^	4.23 ± 0.19 ^a^	1.66 ± 0.01 ^c^	68.88 ± 0.66 ^c^
	FV-CA < 300 kD	21.72 ± 0.44 ^e,f,g^	1.37 ± 0.05 ^e,f^	2.96 ± 0.56 ^a^	4.06 ± 0.01 ^f^
	FV-CA < 100 kD	22.00 ± 1.8 ^e,f,g^	1.14 ± 0.09 ^f^	2.34 ± 0.51 ^a,b,c^	N.F.
	FV-CA < 50 kD	18.76 ± 0.85 ^f,g,h,i^	2.11 ± 0.13 ^d.e,f^	2.13 ± 0.16 ^b,c^	N.F.
	FV-CA < 10 kD	19.28 ± 0.70 ^f,g,h,i^	1.82 ± 0.07 ^d,e,f^	2.43 ± 0.22 ^a,b^	N.F.
	FV-CA < 5 kD	12.93 ± 0.25 ^i^	1.50 ± 0.06 ^e,f^	0.54 ± 0.02 ^d^	N.F.
	FV-HCL > 300 kD	63.59 ± 5.70 ^c^	2.39 ± 0.09 ^c,d,e^	1.71 ± 0.34 ^b,c^	47.49 ± 0.08 ^d^
	FV-HCL < 300 kD	26.66 ± 0.36 ^e^	1.74 ± 0.10 ^d,e,f^	2.27 ± 0.01 ^a,b,c^	1.82 ± 0.01 ^f,g^
	FV-HCL < 100 kD	23.20 ± 1.92 ^e,f^	1.53 ± 0.08 ^e,f^	2.18 ± 0.22 ^b,c^	N.F.
	FV-HCL < 50 kD	20.65 ± 0.72 ^f,g,h^	2.37 ± 0.02 ^c,d,e^	2.04 ± 0.25 ^b,c^	N.F.
	FV-HCL < 10 kD	21.24 ± 0.83 ^e,f,g,h^	2.10 ± 0.08 ^d,e,f^	2.35 ± 0.31 ^a,b,c^	N.F.
	FV-HCL < 5 kD	14.35 ± 0.86 ^h,i^	1.57 ± 0.09 ^d,e,f^	0.75 ± 0.07 ^d^	N.F.

^a–i^ Different letters indicate a statistical difference (*p* < 0.05). N.F.: not found.

**Table 2:** Chemical composition of fucoidan extracted from *A. nodosum.*

Fucoidan Obtained from Various Process	Sample	Fucoidan Content (%)	Total Glucan (mg Total Glucans/100 mg Dried Extract)	Protein (%)	Molecular Weight (kDa)
Crude fucoidan (with alcohol precipitation)	AN-CA-CF	59.82 ± 2.62 ^b^	0.82 ± 0.08 ^h^	0.29 ± 0.00 ^f^	82.14 ± 3.69 ^b^
	AN-HCL-CF	81.20 ± 1.05 ^a^	0.79 ± 0.15 ^h^	0.27 ± 0.00 ^f^	76.25 ± 1.71 ^c^
FD_fucoidan (without alcohol precipitation)	AN-CA-FD	14.59 ± 0.17 ^f,g,h^	1.67 ± 0.18 ^f,g^	0.75 ± 0.03 ^e,f^	102.13 ± 5.03 ^a^
	AN-HCL-FD	12.98 ± 0.18 ^g,h^	2.06 ± 0.20 ^d,e,f^	0.77 ± 0.01 ^e,f^	20.47 ± 1.02 ^e^
MWCO	AN-CA > 300 kD	79.21 ± 2.20 ^a^	3.37 ± 0.07 ^a,b^	2.13 ± 0.01 ^d^	73.49 ± 0.08 ^b^
	AN-CA < 300 kD	20.09 ± 0.89 ^c,d,e^	1.63 ± 0.06 ^g^	4.70 ± 0.04 ^a,b,c^	1.82 ± 0.01 ^f^
	AN-CA < 100 kD	18.26 ± 0.68 ^d,e,f^	1.73 ± 0.07 ^e,f,g^	4.02 ± 0.02 ^c^	N.F.
	AN-CA < 50 kD	17.02 ± 0.40 ^e,f,g^	2.20 ± 0.06 ^d,e,f,g^	3.89 ± 0.03 ^c^	N.F.
	AN-CA < 10 kD	20.41 ± 0.30 ^c,d,e^	2.24 ± 0.02 ^d,e,f,g^	5.17 ± 0.84 ^a^	N.F.
	AN-CA < 5 kD	11.85 ± 0.97 ^h^	1.78 ± 0.11 ^e,f,g^	1.72 ± 0.08 ^d^	N.F.
	AN-HCL > 300 kD	80.70 ± 1.13 ^a^	2.43 ± 0.02 ^c,d,e^	1.74 ± 0.04 ^d^	52.25 ± 1.67 ^d^
	AN-HCL < 300 kD	20.77 ± 1.76 ^c,d,e^	2.79 ± 0.10 ^b,c,d^	4.17 ± 0.01 ^b,c^	1.82 ± 0.01 ^f^
	AN-HCL < 100 kD	20.79 ± 0.44 ^c,d,e^	2.38 ± 0.24 ^c,d^	5.26 ± 0.28 ^a^	N.F.
	AN-HCL < 50 kD	23.41 ± 0.07 ^c^	3.38 ± 0.12 ^a^	4.98 ± 0.94 ^a,b^	N.F.
	AN-HCL < 10 kD	21.29 ± 0.64 ^c,d^	3.40 ± 0.10 ^a^	5.44 ± 0.57 ^a^	N.F.
	AN-HCL < 5 kD	12.24 ± 0.15 ^h^	3.06 ± 0.14 ^a,b,c^	1.46 ± 0.03 ^d,e^	N.F.

^a–h^ Different letters indicate a statistical difference (*p* < 0.05). N.F.: not found.

**Table 3:** Antioxidant activities of fucoidans extracted from *F. vesiculosus* and *A. nodosum.*

Sample	DPPH (%)	FRAP (μM Trolox Equivalents per mg Extract)
FV-CA-CF	62.19 ± 0.99 ^e^	5.54 ± 0.15 ^d^
AN-CA-CF	76.78 ± 1.438 ^a,b^	4.08 ± 0.03 ^e^
FV-HCL-CF	65.44 ± 1.29 ^d,e^	9.97 ± 0.28 ^a^
AN-HCL-CF	80.28 ± 1.16 ^a^	4.68 ± 0.10 ^d,e^
FV-CA > 300 kD	62.80 ± 1.15 ^e^	7.27 ± 0.05 ^c^
FV-CA < 300 kD	61.99 ± 1.35 ^e^	4.67 ± 0.14 ^d,e^
FV-HCL > 300 kD	69.91 ± 1.29 ^c,d^	6.70 ± 0.12 ^c^
FV-HCL < 300 kD	60.97 ± 0.86 ^e^	2.65 ± 0.02 ^f^
AN-CA > 300 kD	80.08 ± 0.92 ^a^	9.86 ± 0.15 ^a^
AN-CA < 300 kD	71.95 ± 1.43 ^b,c,d^	3.94 ± 0.04 ^e^
AN-HCL > 300 kD	72.76 ± 1.16 ^b,c^	9.67 ± 0.15 ^a,b^
AN-HCL < 300 kD	66.87 ± 0.92 ^c,d,e^	8.89 ± 0.22 ^b^
Fucoidan sigma standard	84.91 ± 0.96	11.49 ± 0.06
Vitamin C standard	77.64 ± 1.16	

^a–f^ Different letters indicate a statistical difference (*p* < 0.05).

## Data Availability

The data presented in this study are available on request from the corresponding author.
